# D-TRP(8)-γMSH Prevents the Effects of Endotoxin in Rat Skeletal Muscle Cells through TNFα/NF-KB Signalling Pathway

**DOI:** 10.1371/journal.pone.0155645

**Published:** 2016-05-13

**Authors:** Ana Belén Gómez-SanMiguel, María Ángeles Villanúa, Ana Isabel Martín, Asunción López-Calderón

**Affiliations:** Department of Physiology, Facultad de Medicina, Universidad Complutense de Madrid, 28040 Madrid, Spain; University of Louisville School of Medicine, UNITED STATES

## Abstract

Sepsis induces anorexia and muscle wasting secondary to an increase in muscle proteolysis. Melanocyte stimulating hormones (MSH) is a family of peptides that have potent anti-inflammatory effects. Melanocortin receptor-3 (MC3-R) has been reported as the predominant anti-inflammatory receptor for melanocortins. The aim of this work was to analyse whether activation of MC3-R, by administration of its agonist D-Trp(8)-γMSH, is able to modify the response of skeletal muscle to inflammation induced by lipopolysaccharide endotoxin (LPS) or TNFα. Adult male rats were injected with 250 μg/kg LPS and/or 500 μg/kg D-Trp(8)-γMSH 17:00 h and at 8:00 h the following day, and euthanized 4 hours afterwards. D-Trp(8)-γMSH decreased LPS-induced anorexia and prevented the stimulatory effect of LPS on hypothalamic IL-1β, COX-2 and CRH as well as on serum ACTH and corticosterone. Serum IGF-I and its expression in liver and *gastrocnemius* were decreased in rats injected with LPS, but not in those that also received D-Trp(8)-γMSH. However, D-Trp(8)-γMSH was unable to modify the effect of LPS on IGFBP-3. In the *gastrocnemius* D-Trp(8)-γMSH blocked LPS-induced decrease in pAkt, pmTOR, MHC I and MCH II, as well as the increase in pNF-κB(p65), FoxO1, FoxO3, LC3b, Bnip-3, Gabarap1, atrogin-1, MuRF1 and in LC3a/b lipidation. In L6 myotube cultures, D-Trp(8)-γMSH was able to prevent TNFα-induced increase of NF-κB(p65) phosphorylation and decrease of Akt phosphorylation as well as of IGF-I and MHC I expression. These data suggest that MC3-R activation prevents the effect of endotoxin on skeletal wasting by modifying inflammation, corticosterone and IGF-I responses and also by directly acting on muscle cells through the TNFα/NF-κB(p65) pathway.

## Introduction

Sepsis, like many other inflammatory conditions, induces cachexia, which increases mortality and morbidity [1]. Inflammatory cachexia is associated with anorexia, fatigue, and muscle wasting. Skeletal muscle wasting in sepsis is mainly due to activation of muscle proteolysis, rather than to a decrease in muscle protein synthesis [2]. The ubiquitin-proteasome proteolytic pathway is increased in sepsis, and two E3 ubiquitin ligases, muscle ring-finger-1 (MuRF1) and atrogin-1, are sensitive markers for muscular atrophy [3, 4]. Recently, autophagy has also been involved in sepsis-induced muscle wasting [5] Protein kinase B (Akt)/ Forkhead box protein O (FoxO) and nuclear factor kappa-light-chain-enhancer of activated B cells (NF-κB) are cellular pathways and transcription factors that are clearly involved in muscle atrophy in sepsis and activate the ubiquitin-proteasome system and autophagy [6, 7].

There are multiple systemic factors responsible for inflammation-induced muscle wasting. Among the main regulators of muscle mass, inflammatory signalling plays a critical role in regulating the anabolic/catabolic balance in muscle via activation of the hypothalamic-pituitary-adrenal axis, through glucocorticoid release [8, 9]. In addition, changes in insulin-like growth factor I (IGF-I) and in the release of cytokines or other inflammatory mediators have also been proposed as stressors that can trigger skeletal muscle wasting [7].

Melanocyte stimulating hormones (α, β, and γMSH) are a family of peptide hormones that regulate skin pigment cells and affect a range of other processes in the body, such as decreasing inflammation [10]. Peripheral αMSH treatment decreases the acute inflammatory response to endotoxin and increases survival in experimental models of septic shock [10, 11]. We have previously reported that systemic αMSH administration blunts skeletal muscle response to endotoxin and to chronic arthritis by exerting anti-inflammatory and antiproteolytic activities [12, 13]. The potent anti-inflammatory effects of αMSH have been shown to be mediated through blockade of NF-κB activation and decreasing the release of pro-inflammatory cytokines [13–15]. Among the types of αMSH receptors, MC3-R and MC4-R have been shown to have anti-inflammatory effects [16, 17]. The MC3-R is abundantly distributed in both the brain and in the periphery, whereas MC4-R is primarily found in the brain [18]. MC3-R activation by its agonist, γMSH, suppresses cellular and systemic inflammation in response to pro-inflammatory stimuli [19]. In addition, it has been reported that MC3-RKO mice suffer enhanced anorexia and weight loss with LPS challenge and with tumour growth [20]. Furthermore, administration of a MC3-R agonist prevents muscle wasting induced by experimental arthritis by down-regulating atrogenes and autophagy [21].

The aim of this work was to elucidate whether the anti-cachectic effects of αMSH in endotoxin-injected rats is mediated by activation of its MC3-R. MC3-R has been reported in skeletal muscle [18]. Therefore, the possible direct action of a MC3-R agonist on skeletal muscle cells has also been tested. To this end, we administered D-Trp8-γMSH to adult male rats. The D-Trp8-γMSH analogue is a potent and selective MC3-R agonist, with a 100-fold selectivity for the MC3-R relative to the MC4-R [22]. Herein, we show that activation of MC3-R ameliorates LPS-induced anorexia and muscle proteolysis by decreasing inflammation as well as the changes in glucocorticoid and IGF-I release, but also through a direct action on muscle cells.

## Material and Methods

### Animals

Male Wistar rats weighing 200 g were purchased from Harlan (Barcelona, Spain). Rats were housed 2 per cage, and maintained under standardized conditions of temperature (20–22°C) and light (lights on from 7:30 am to 7:30 pm). Rats were quarantined for at least 1 week before any experimental use. The physical conditions of the animals were checked once every 2 days All efforts were taken to minimize animal suffering. The procedures followed the guidelines recommended by the EU for the care and use of laboratory animals, and were approved by the Complutense University Animal Care Committee (approval ID: CEA-UCM 16/12).

Rats were randomly assigned to the following treatment groups of 10 rats and fed ad libitum: (1) control, i.p. injected with 250 μl sterile saline, (2) control + with 500 μg/kg D-Trp(8)-γMSH (American Peptide, Sunnyvale, CA, USA) dissolved in saline, (3) LPS, i.p. injected with 250 μg/kg LPS (serotype 055:B5, Sigma Chemical Co.), and (4) LPS + D-Trp(8)-γMSH, which was simultaneously i.p. injected with both compounds in 250 μl saline. As LPS decreases food intake, a pair-fed (PF) group was added; it was injected with saline and received the same amount of food eaten by the group of rats injected with LPS. Rats received treatment at 17:00 h and at 08:00 h the following day. This LPS administration protocol was shown to decrease levels of IGF-I in serum and liver, and to increase MuRF1 and atrogin-1 in skeletal muscle [23, 24]. None of the animals became ill or died prior to the experimental endpoint. All animals were euthanized by decapitation at 12:00 h, 19 h after the first, and 4 h after the second LPS and/or D-Trp(8)-γMSH injection. Trunk blood was collected, allowed to clot, and the serum was stored at −20°C for IGF-I, insulin-like growth factor-binding protein 3 (IGFBP-3), adrenocorticotropin hormone (ACTH), corticosterone and nitrite assays. The medial basal hypothalami were dissected as previously described [25], quickly frozen in liquid nitrogen and stored at -80°C for RNA isolation. Liver and *gastrocnemius* muscle were removed, frozen immediately in liquid nitrogen, and stored at -80°C for isolation of mRNA or proteins.

### Myotube cultures

Myoblasts derived from rat skeletal muscle (L6 cells; ATCC, Manassas, Virginia, USA) were cultured in 6-well plates containing Dulbecco’s modified Eagle’s medium (DMEM) supplemented with 10% heat-inactivated fetal bovine serum (FBS), 1% penicillin-streptomycin at 37°C under a humidified 5%CO_2_/95%O_2_ atmosphere. When myoblasts were approximately 75% confluent, myotube differentiation was initiated by replacing the growth medium with differentiation medium: DMEM supplemented with 1% FBS. Differentiation was allowed to continue for 7 days before experimentation.

Fully differentiated L6 myotubes were treated and incubated for 24 h with recombinant rat TNFα (PeproTech, Princeton, New Jersey, USA) (10 μg/ml) and/or D-Trp(8)-γMSH (American Peptide, Sunnyvale, CA, USA) (0, 50 and 200 nM) or DMEM alone. At this concentration (10μg/ml) TNFα induces activation of NF-kB and down-regulation of IGF-I and Akt in C2C12 cells [26]. At the end of the incubation period, total RNA or proteins from cells were extracted.

### RNA extraction and real-time PCR

*Gastrocnemius* or liver (100 mg) was homogenized, and total RNA was extracted using UltraspecTM (Biotecx Laboratories Inc. Houston, Texas, USA), following the manufacturer’s protocol. Total RNA was extracted from myotube cultures using REAL TOTAL RNA, C.E. (DURVIZ S.L., Valencia, Spain) according to the protocol supplied by the manufacturer. Total RNA was dissolved in 0.1% SDS diethylpyrocarbonate-treated water and quantified at 260 nm. The final concentration of RNA was determined (260 nm) with a BioPhotometer (Eppendorf, Germany), and the integrity of the RNA was confirmed by agarose gel electrophoresis. First-strand cDNA synthesis was performed using 1 μg of total RNA with a Quantiscript Reverse Transcription kit (Qiagen, Valencia, CA, USA).

Real-time PCR for quantification of mRNA was performed on a SmartCycler® (Cepheid, Sunnyvale, CA, USA) using a SYBR-Green protocol on the fluorescence temperature cycler. Each real-time PCR reaction consisted of 10 ng total RNA equivalents,1x Takara SYBR Green Premix Ex Taq (Takara BIO INC, Otsu, Shiga, Japan), and 300 nM forward and reverse primers in a reaction volume of 25.5 μl. Primers for real-time PCR (Table 1) were obtained from Roche (Madrid, Spain). The thermal cycling profile consisted of a pre-incubation step at 95°C for 10 s followed by 40 cycles of 95°C denaturation steps for 15 s, 60°C annealing steps for 30 s, and 72°C extension steps for 30 s. Results were expressed relative to the control animals injected with saline, where the relative mRNA abundance had been arbitrarily set to 1, using cycle threshold 2(ΔΔCT) method, with 18S and HPRT as reference genes. PCR products were separated using agarose gel electrophoresis to confirm the product presence and size.

**Table 1:** Primers for real-time PCR.

Gene	Forward Primer (5' to 3')	Reverse Primer (5' to 3')	Product bp
**18S**	GGTGCATGGCCGTTCTTA	TCGTTCGTTATCGGAATTAACC	60
**HPRT**	CTCATGGACTGATTATGGACAGGAC	GCAGGTCAGCAAAGAACTTATAGCC	122
**COX-2**	ACCAACGCTGCCACAACT	GGTTGGAACAGCAAGGATTT	118
**TNF-α**	TGAACTTCGGGGTGATCG	GGGCTTGTCACTCGAGTTTT	122
**IL-1β**	GCTGTGGCAGCTACCTATGTCTTG	AGGTCGTCATCATCCCACGAG	120
**CRH**	CGCAGCCGTTGAATTTCTTG	GCGGGACTTCTGTTGAGG	112
**IGF-I**	GCTATGGCTCCAGCATTCG	TCCGGAAGCAACACTCATCC	62
**IGFBP-3**	GGAAAGACGACGTGCATTG	GCGTATTTGAGCTCCACGTT	78
**LC3b**	CAGGTTGCCTAGCAGAGGTC	TGTCCTATACACCTGACCTGTTTC	67
**Bnip-3**	CAGAGCGGGGAGGAGAAC	GAAGCTGGAACGCTGCTC	80
**Gabarap1**	TATCCCTCCCACCAGTGCTA	AAATAGTCTTCCTCATGGTTGTCC	63
**atrogin-1**	GAACAGCAAAACCAAAACTCAGTA	GCTCCTTAGTACTCCCTTTGTGAA	74
**MuRF-1**	TGTCTGGAGGTCGTTTCCG	ATGCCGGTCCATGATCACTT	58
**MHC I**	CCTGCAGCTCCAAGTTCAGT	ATCAGCTGGTCGCATCTTTC	69
**MHC IIa**	CCATATATTTTATCAAATCACATCCAA	GGTGATCAGCAGCATTTCG	64

### Western blot

*Gastrocnemius* and myotubes were homogenized in RIPA buffer (10μl/ mg) with protease inhibitor cocktail, sodium deoxycolate 12.5 mM, phenylmethane sulfonyl fluoride 100 mM, sodium orthovanadate 12.5 mM and with phosphatase inhibitors (Sigma-Aldrich, Madrid, Spain). The homogenate was later centrifuged at 13000 rpm at 4°C for 30 min to remove tissue debris. Protein concentration was determined using the Bradford protein assay with bovine serum albumin as standard. The protein extract was boiled for 5 min with a 1:1 volume of Laemmli loading buffer. Proteins (100 μg from *gastrocnemius* or 20 μg from myotubes) were resolved by electrophoresis on 14% polyacrylamide gels under reducing conditions, and then transferred onto nitrocellulose membranes that were blocked by incubation in 5% non-fat dry milk, 0.1% Tween (Sigma-Aldrich, Madrid, Spain), in Tris-buffered saline. Ponceau-S staining was performed to ensure equal transfer prior to blocking. Membranes were probed overnight at 4°C sequentially with antibodies against pAktSer(473) (1:1000, Cell Signaling Technology, antibody ID: AB_2315049), Akt (1:2000, Santa Cruz Biotechnology, antibody ID: AB_671714), p-mammalian target of rapamycin (pmTOR) (1:750, Cell Signaling Technology, antibody ID: AB_330970), mTOR (1:1000, Cell Signaling Technology, antibody ID: AB_10695460), microtubule-associated protein-1 light chain 3 (LC3A/B (D3U4C) XP® Rabbit mAb) (1:1000, Cell Signaling Technology, antibody ID: 12741), pNF-κBp65Ser(536) (1:1000, Cell Signaling Technology, antibody ID: AB_331284, clon 7F1), pNF-κBp65Ser(276) (1:1000, Santa Cruz Biotechnology, antibody ID: AB_1128534), NF-κBp65 (C20) (1:1000, Santa Cruz Biotechnology, antibody ID: AB_632037, clon C-20), pFoXO1Ser(276) (1:750, Cell Signaling Biotechnology, antibody ID: AB_10827635), FoXO1 (1:1000, Santa Cruz Biotechnology, antibody ID: AB_640607), pFoxO3a (1:500, Santa Cruz Biotechnology, antibody ID: AB_653226), FoxO3a (D19A7) (1:750, Cell Signaling Technology, supplier Catalog no: #12829), atrogin-1 (1:1000, Santa Cruz Biotechnology, antibody ID: AB_2104267, clon H-300), MuRF1 (1:1000, Santa Cruz Biotechnology, antibody ID: AB_2287871, clon H-145) and α-tubulin (1:5000, Sigma-Aldrich, antibody ID: T5168); with stripping of membranes, using stripping buffer (Restore Western Blot Stripping Buffer, Thermo-scientific Rockford, Il, USA) before each new antibody. Membranes were then incubated for 90 min in the appropriate secondary antibody conjugated to horseradish peroxidase (anti-mouse IgG (Amersham Biosciences, Little Chalfont, UK); anti-rabbit IgG (GE Healthcare, Madrid, Spain); anti-goat IgG (Santa Cruz Biotechnology), and peroxidase activity was detected using enhanced chemiluminescent reagent (Amersham Biosciences, Little Chalfont, UK). Band intensities were quantified by densitometry using a PC-Image VGA24 program for Windows. The density of the protein band in each lane was expressed as the percentage of the mean density of control rats, after load normalization using α-tubulin.

### Serum IGFBP-3 measurement

Serum concentrations of IGFBP-3 were measured by ligand blot. Two μl of serum were diluted in sample buffer and boiled 2 min at 90°C, loaded onto 1% SDS-12.5% polyacrylamide gels, and electrophoresed under non-reducing conditions. Proteins were transferred onto nitrocellulose sheets (HybondTM-C extra, Amersham, UK). The membranes were dried and blocked for 1 h with 5% non-fat dry milk and 0.1% Tween (Sigma), in Tris-buffered saline. Membranes were probed overnight at 4°C with ^125^I-labelled IGF-I (1.5 x 10^6^ cpm/ml). The nitrocellulose sheets were then washed, dried and exposed at -80°C to X-ray film (Kodak X-Omat AR, Eastman Kodak, Rochester, NY, USA) and to two intensifying screens for 1–4 days according to the signal obtained. The film signals were quantified by densitometry using a PC-Image VGA24 program for Windows. The density of the IGFBP-3 band in each lane was expressed as the percentage of the mean density of sera from its respective control rats.

### Serum IGF-I, ACTH, corticosterone and nitrite measurements

Serum IGF-I was measured using the anti-serum to human IGF-I (UB2-495) from Dr Underwood and Dr Van Wik, which is distributed by the National Institute of Diabetes and Digestive and Kidney Diseases (NIDDK) Hormone Distribution Programme through the National Hormone and Pituitary Programme. Levels of IGF-I were expressed in terms of rat IGF-I from Gropep Ltd. (Adelaide, Australia). The intra-assay coefficient of variation was 8%. All samples from the same experiment were run in the same assay.

Serum ACTH and corticosterone was analysed by a commercial kit from MP Biomedicals, LLC (Orangeburg, NY, USA), following the manufacturer’s protocols.

Nitrite + nitrate concentrations in serum were measured by a modified method of Griess assay. Serum was deproteinized to reduce turbidity by centrifugation through a 30 kDa molecular weight filter using a Centrifree Micropartition Device with a YM-30 ultrafiltration membrane (Amicon Division, Millipore Corporation, Bedford, TX, USA), at 15000 rpm for 1 h at 37°C for 300 μL samples. One hundred μL of filtrated serum was mixed with 100 μl of vanadium chloride and was quickly followed by the addition of the Griess reagents. The determination was performed after incubation at 37°C for 30 min. The absorbance was measured at 540 nm. Nitrite and nitrate concentrations were calculated using a NaNO_2_ standard curve.

### Statistical analysis

Statistics were computed using the statistics program STATGRAPHICS plus for Windows. Data are presented as mean ± S.E.M. and differences among experimental groups were analysed by one-way analysis of variance. Post-hoc comparisons were made by using subsequent LSD multiple range tests. Statistical significance was set at P < 0.05.

## Results

### Body weight, food intake and liver inflammation

**Fig 1:**
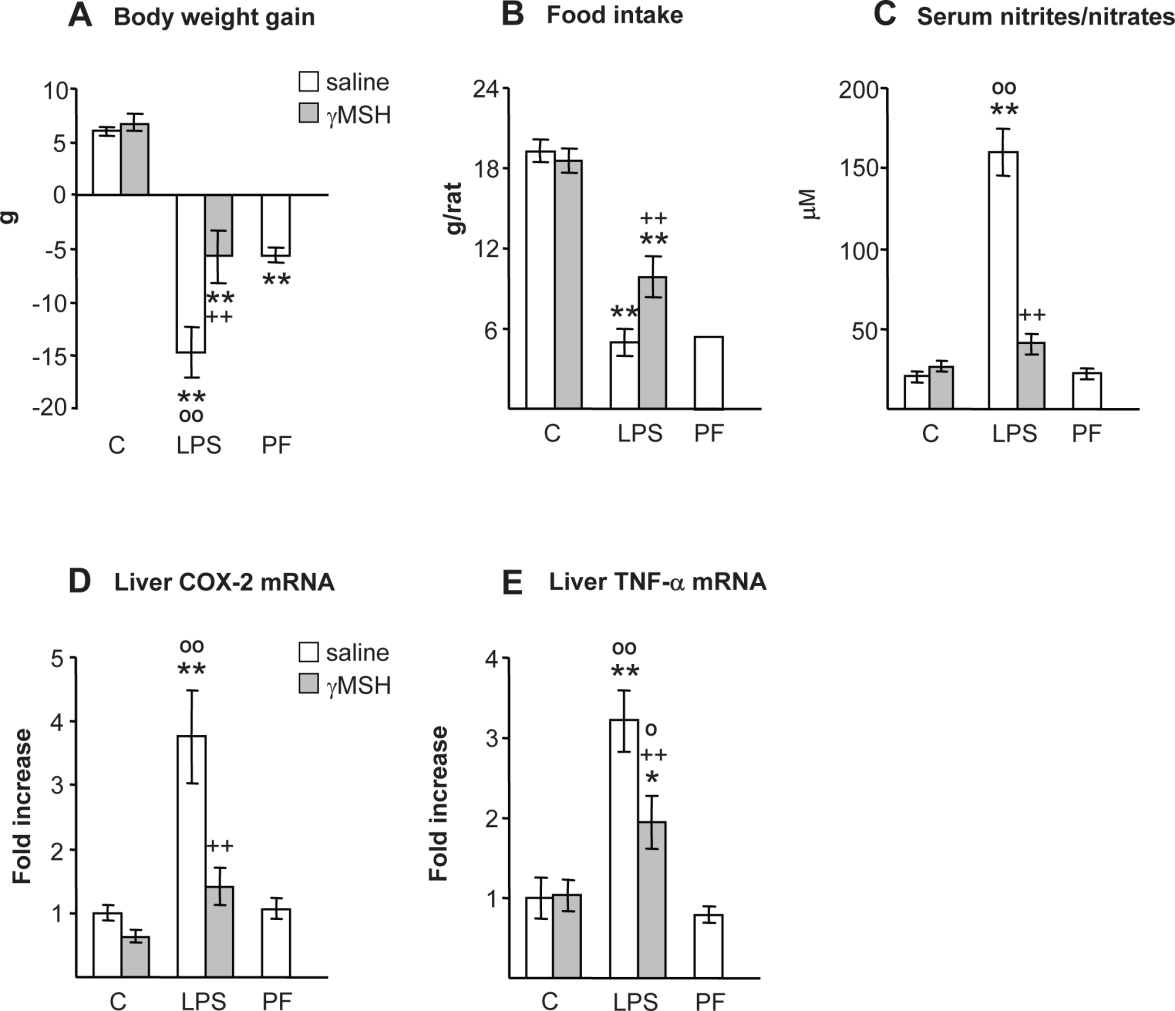
Effect of D-Trp(8)-γMSH (γMSH) treatment (500 μg/kg i.p.) on: body weight gain (A), food intake (B), serum nitrite + nitrate levels (C), liver COX-2 mRNA (D) and liver TNFα mRNA (E) in control rats or in rats treated with LPS (250 μg/kg i.p.). PF = pair-fed rats. D-Trp(8)-γMSH treatment decreased the inhibitory effect of LPS administration on body weight and food intake as well as the stimulatory effect of LPS on serum concentration of nitrites + nitrates, liver TNFα and COX-2 mRNA (P<0.01). Results are expressed as means ± SE for 8–10 rats per group. *P< 0.05 and **P< 0.01, vs. their respective control group. ++P<0.01 vs. LPS-saline, °P<0.05, °°P<0.01 vs. PF. LSD multiple comparison test, following one-way ANOVA.

As expected, LPS injection decreased body weight gain compared with control and pair-fed rats (P<0.01, Fig 1A). Administration of D-Trp(8)-γMSH attenuates LPS-induced decreases in body weight (P<0.01), where the change in body weight in this group was similar to that of pair-fed rats. LPS also decreased food intake in both groups of rats, treated with either saline or D-Trp(8)-γMSH, but the decrease was lower in the rats treated with D-Trp(8)-γMSH (P<0.01, Fig 1B). LPS increased serum nitrite + nitrate and the expression of COX-2 in the liver (P<0.01, Fig 1C and 1D) in the rats treated with saline, but not in those treated with D-Trp(8)-γMSH. Liver TNFα mRNA was also significantly increased by LPS injection (P<0.01 Fig 1E), and D-Trp(8)-γMSH administration attenuated LPS-induced increase in liver TNFα (P<0.01).

### D-Trp(8)-γMSH suppressed LPS-induced hypothalamic inflammation and activation of the adrenal axis

**Fig 2:**
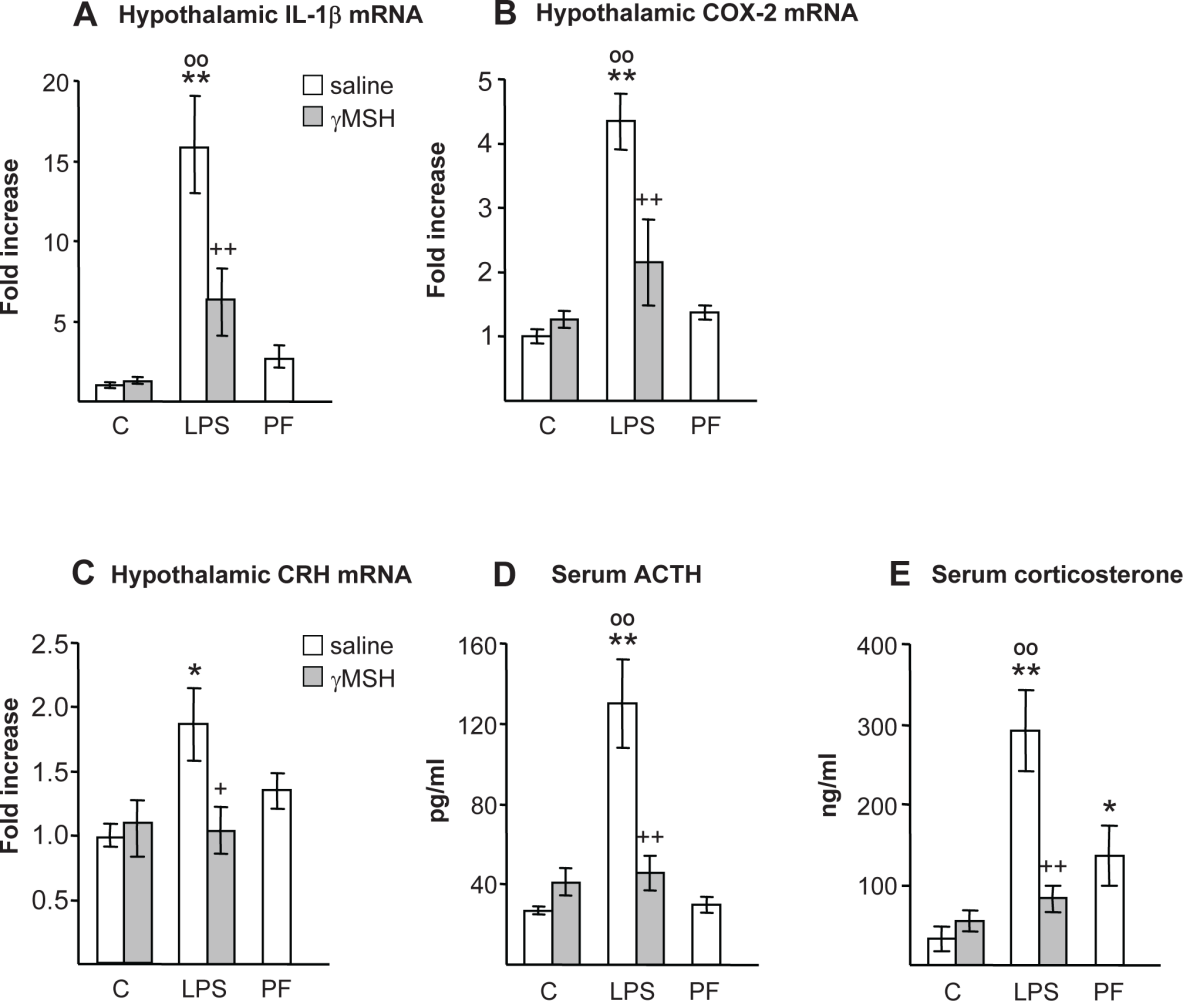
Effect of D-Trp(8)-γMSH (γMSH) (500 μg/kg i.p.) administration on hypothalamic mRNA expression of: IL-1β (A), COX-2 (B) and CRH (C), and on serum concentrations of ACTH (D) and corticosterone (E) in control (C) and in LPS-injected (250 μg/kg) rats. PF = pair-fed rats. Each bar represents the mean ± SE for n = 7–10. mRNA expression was quantified using real-time RT-PCR and is presented as the increase of the mean value in control rats treated with saline. LPS injection increased hypothalamic IL-1β, COX-2 and CRH mRNA levels as well as serum ACTH and corticosterone levels (P<0.01) in rats injected with saline, but not in rats injected with γMSH. *P< 0.05 and **P< 0.01, vs. their respective control group. +P<0.05, ++P<0.01 vs. LPS-saline, °°P<0.01 vs. PF. LSD multiple comparison test, following one-way ANOVA.

LPS injection also triggered hypothalamic inflammation in the rats treated with saline, since it increased hypothalamic interleukin-1β (IL-1β) and COX-2 mRNA (P<0.01, Fig 2A and 2B), but it was not triggered in those treated with D-Trp(8)-γMSH. Pair-feeding rats did not modify hypothalamic IL-1β or COX-2 mRNA levels. Hypothalamic corticotrophin releasing hormone (CRH) mRNA was increased in the rats injected with LPS (P<0.05, Fig 2C), whereas D-Trp(8)-γMSH treatment blocked the effect of LPS on hypothalamic CRH. Similarly, LPS injection increased serum concentrations of ACTH and corticosterone (P<0.01, Fig 2D and 2E), and D-Trp(8)-γMSH administration prevented the stimulatory effect of LPS on these hormones. Pair-feeding rats increased serum concentration of corticosterone (P<0.05), and tended to increase hypothalamic CRH mRNA, but this increase was not significant.

### IGF-I and IGFBP-3

**Fig 3:**
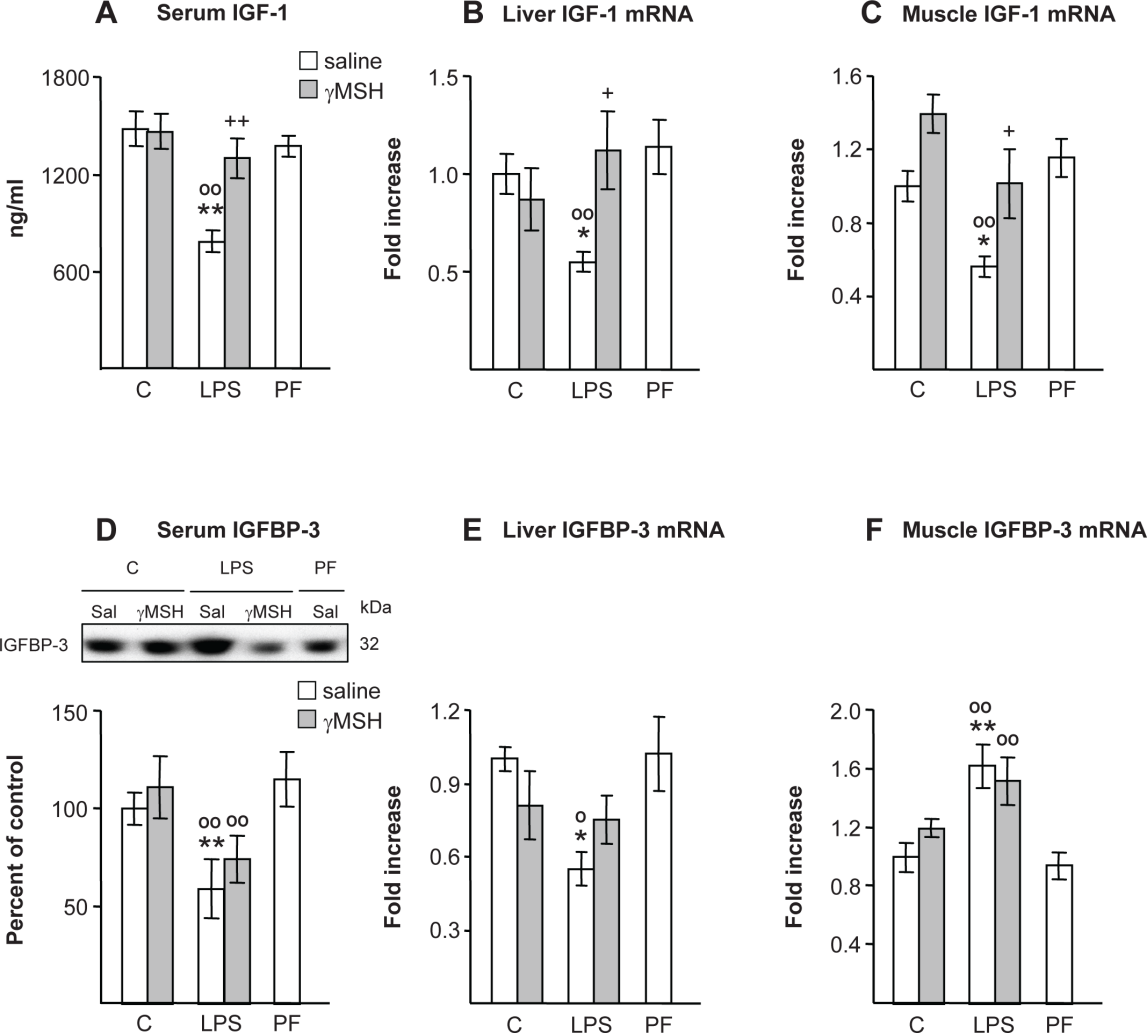
Effect of D-Trp(8)-γMSH (γMSH) treatment (500 μg/kg i.p.) on: IGF-I and IGFBP-3 levels in serum (A and D) and their mRNA in liver (B and E) and *gastrocnemius* (C and F) in control rats or in rats treated with LPS (250 μg/kg). PF = pair-fed rats. γMSH treatment blocked the inhibitory effect of LPS administration on IGF-I in serum and its mRNA in liver and skeletal muscle. LPS decreased serum IGFBP-3 (P<0.01) and its mRNA in the liver (P<0.05), whereas IGFBP-3 mRNA was increased in muscle by LPS injection (P<0.01). γMSH treatment was unable to modify the effects of LPS on IGFBP-3. mRNA expression was quantified using real-time RT-PCR and is presented as the increase of the mean value in control rats treated with saline. Results are expressed as means ± SE for 6–10 rats per group. *P< 0.05 and **P< 0.01, vs. their respective control group. +P<0.05, ++P<0.01 vs. LPS-saline, °P<0.05, °°P<0.01 vs. PF. LSD multiple comparison test, following one-way ANOVA.

As shown in Fig 3A–3C, LPS decreased serum concentration of IGF-I (P<0.01) as well as IGF-I expression in the liver and in *gastrocnemius* (P<0.05). Pair feeding rats did not modify circulating IGF-I or its expression in liver or skeletal muscle. D-Trp(8)-γMSH administration prevented the inhibitory effect of LPS on serum concentrations of IGF-I, where the rats injected with LPS and D-Trp(8)-γMSH had IGF-I levels similar to those found in their controls or in pair-fed rats. D-Trp(8)-γMSH administration was also able to prevent the inhibitory effect of LPS on IGF-I mRNA levels in liver and *gastrocnemius*. Serum concentration of IGFBP-3 was decreased by LPS injection in rats that were either treated with saline or D-Trp(8)-γMSH (P<0.01, Fig 3D). In the rats treated with saline liver IGFBP-3 mRNA was decreased by LPS (P<0.05, Fig 3E), but muscle IGFBP-3 mRNA was increased by LPS (P<0.01, Fig 3F). The rats treated with LPS and D-Trp(8)-γMSH had IGFBP-3 mRNA levels in liver and muscle similar to those of the rats treated with LPS alone. Pair-feeding rats did not modify IGFBP-3 levels.

### NF-κB(p65), Akt/mTOR and FoxO signalling pathways

**Fig 4:**
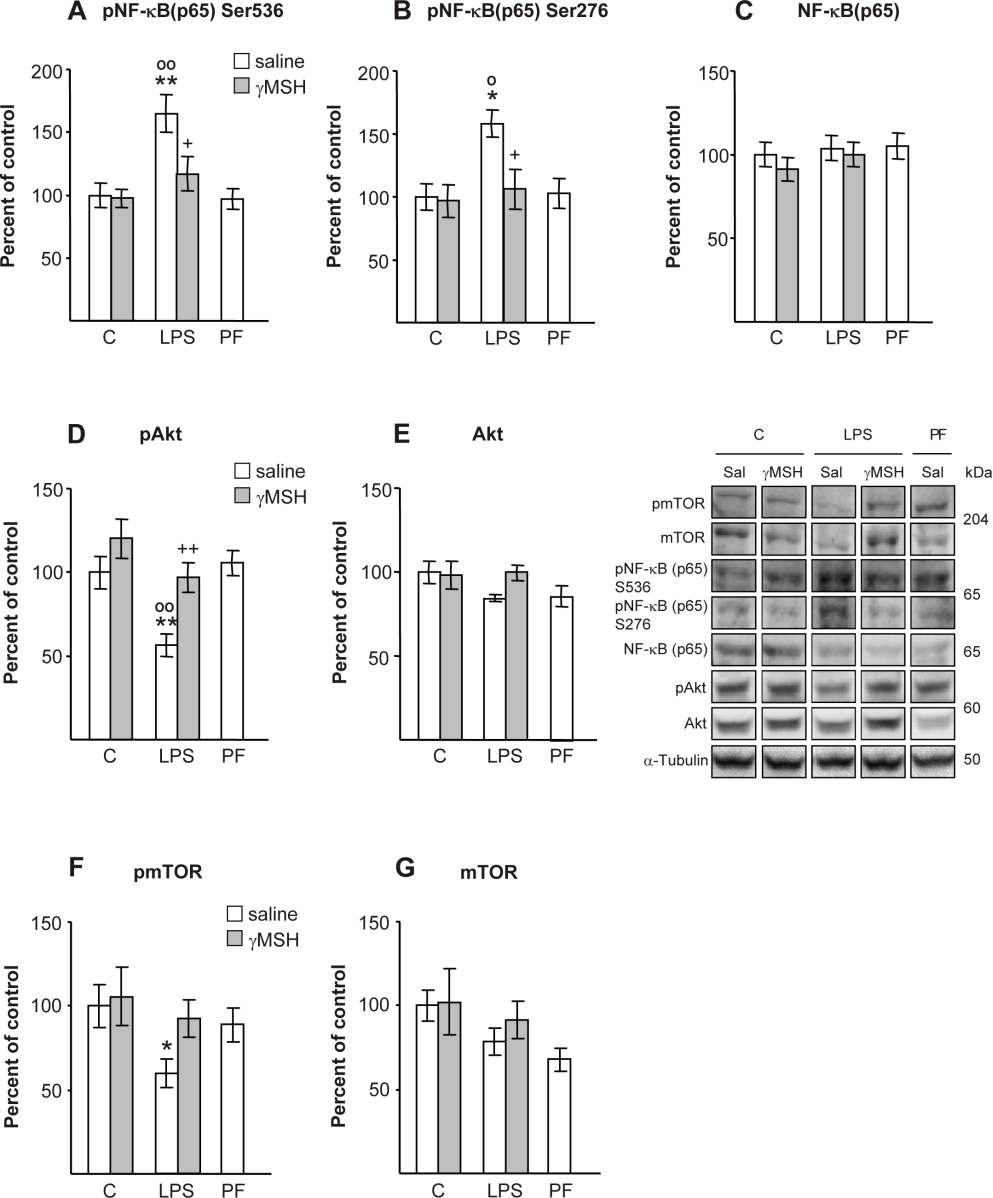
Effect of D-Trp(8)-γMSH (γMSH) treatment (500 μg/kg i.p.) on; phospho-NF-κB(p65)Ser536 (A), phospho-NF-κB(p65)Ser276 (B), NF-κB(p65) (C), phospho-Akt (D), Akt (E), phospho-mTOR (F) and mTOR (G), in *gastrocnemius* muscle of control rats and rats treated with LPS (250 μg/kg). PF = pair-fed rats. Proteins were measured by Western blotting with specific antibodies for total and phosphoprotein and expressed as percentage of the control rats treated with saline. Representative Western blots are shown at the middle right. Boxes with immunoblots represent spliced images based on group and treatment order. LPS increased pNF-κB(p65)Ser536 (P<0.01) and pNF-κB(p65)Ser276 (P<0.05), whereas it decreased pAkt (P<0.01) and pmTOR (P<0.05) in rats treated with saline, but not in those treated with γMSH. Data represent means SE (n = 7–10 rats). *P< 0.05 and **P< 0.01, vs. their respective control group. +P<0.05, ++P<0.01 vs. LPS-saline, °P<0.05, °°P<0.01 vs. PF. LSD multiple comparisons test, following one way ANOVA.

LPS injection increased the phosphorylation of p65 at Ser 536 (P<0.01) and at Ser 276 (P<0.05), in rats treated with saline, but not in those treated with D-Trp(8)-γMSH (Fig 4A–4C). LPS injection did not modify total Akt (Fig 4E), whereas it decreased phosphorylation of Akt to levels lower than those of control or pair-fed rats (P<0.01, Fig 4D). D-Trp(8)-γMSH administration was able to prevent LPS-induced decrease in pAkt (P<0.01). The effects of LPS and D-Trp(8)-γMSH on mTOR activation (Fig 4F and 4G) were similar to those on Akt. LPS decreased phospho-mTOR in rats injected with saline (P<0.05), but not in rats injected with D-Trp(8)-γMSH. Total mTOR was not modified by either of the treatments.

**Fig 5:**
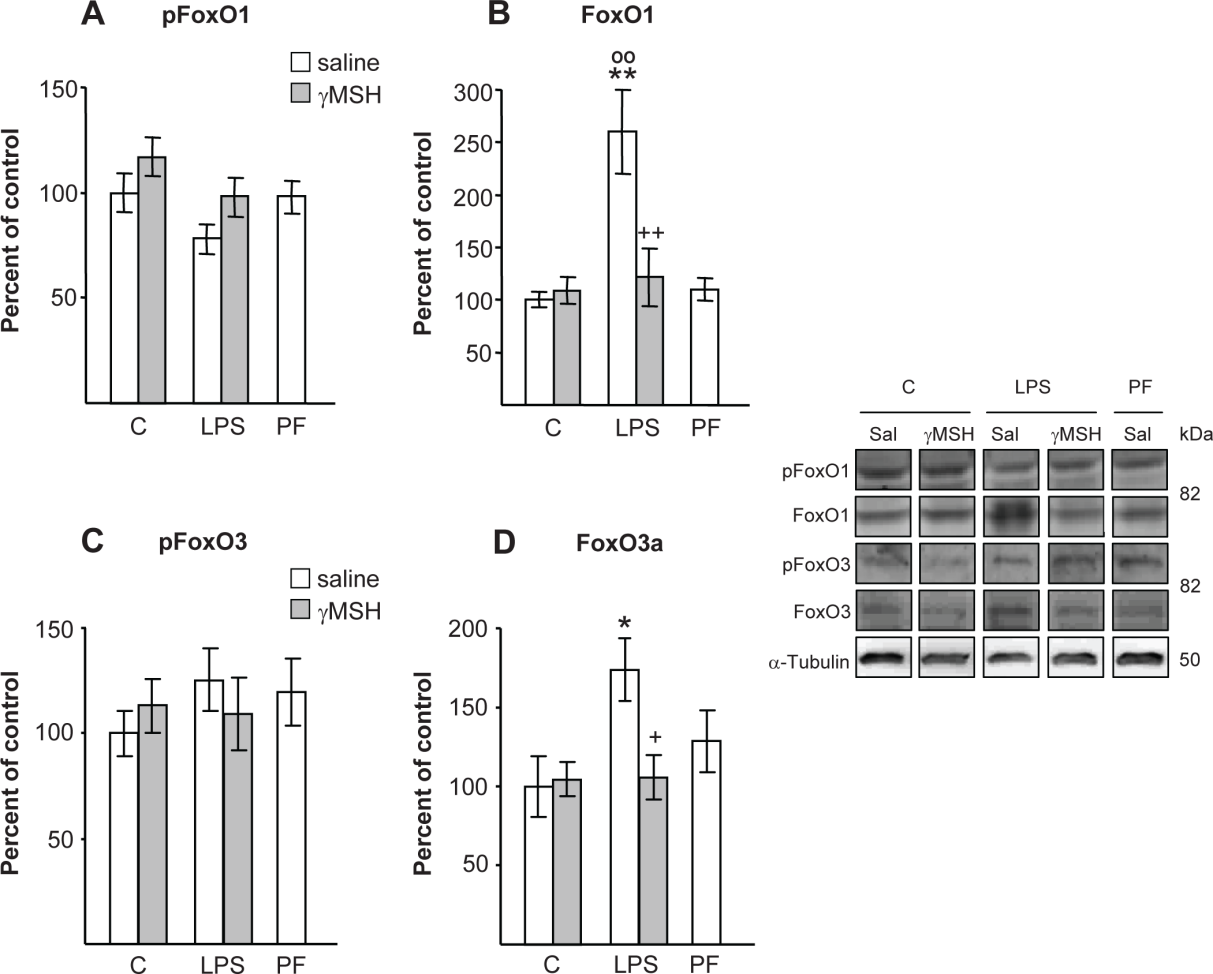
Effect of D-Trp(8)-γMSH (γMSH) treatment (500 μg/kg i.p.) on: phospho-FoxO1 (A), FoxO1 (B), phospho-FoxO3 (C) and FoxO3 (D), in *gastrocnemius* muscle of control rats and rats treated with LPS (250 μg/kg). PF = pair-fed rats. Proteins were measured by Western blotting with specific antibodies for total and phosphoprotein and expressed as percentage of the control rats treated with saline. Representative Western blots are shown at the right. Boxes with immunoblots represent spliced images based on group and treatment order. Data represent means ± SE (n = 7–10 rats). *P< 0.05 and **P< 0.01, vs. their respective control group. +P<0.05, ++P<0.01 vs. LPS-saline, °°P<0.01 vs. PF. LSD multiple comparisons test, following one way ANOVA.

In the rats injected with LPS, FoxO1 and FoxO3 levels in the *gastrocnemius* were increased (Fig 5B and 5D), whereas pFoxO1 and pFoxO3 were not significantly modified (Fig 5A and 5C). D-Trp(8)-γMSH treatment also prevented LPS-induced increase in both FoxO1 (P<0.01) and FoxO3 (P<0.05) levels in the *gastrocnemius*.

### D-Trp(8)-γMSH prevented the increase in autophagic response and in MuRF1 and atrogin-1 levels in muscle after LPS injection

**Fig 6:**
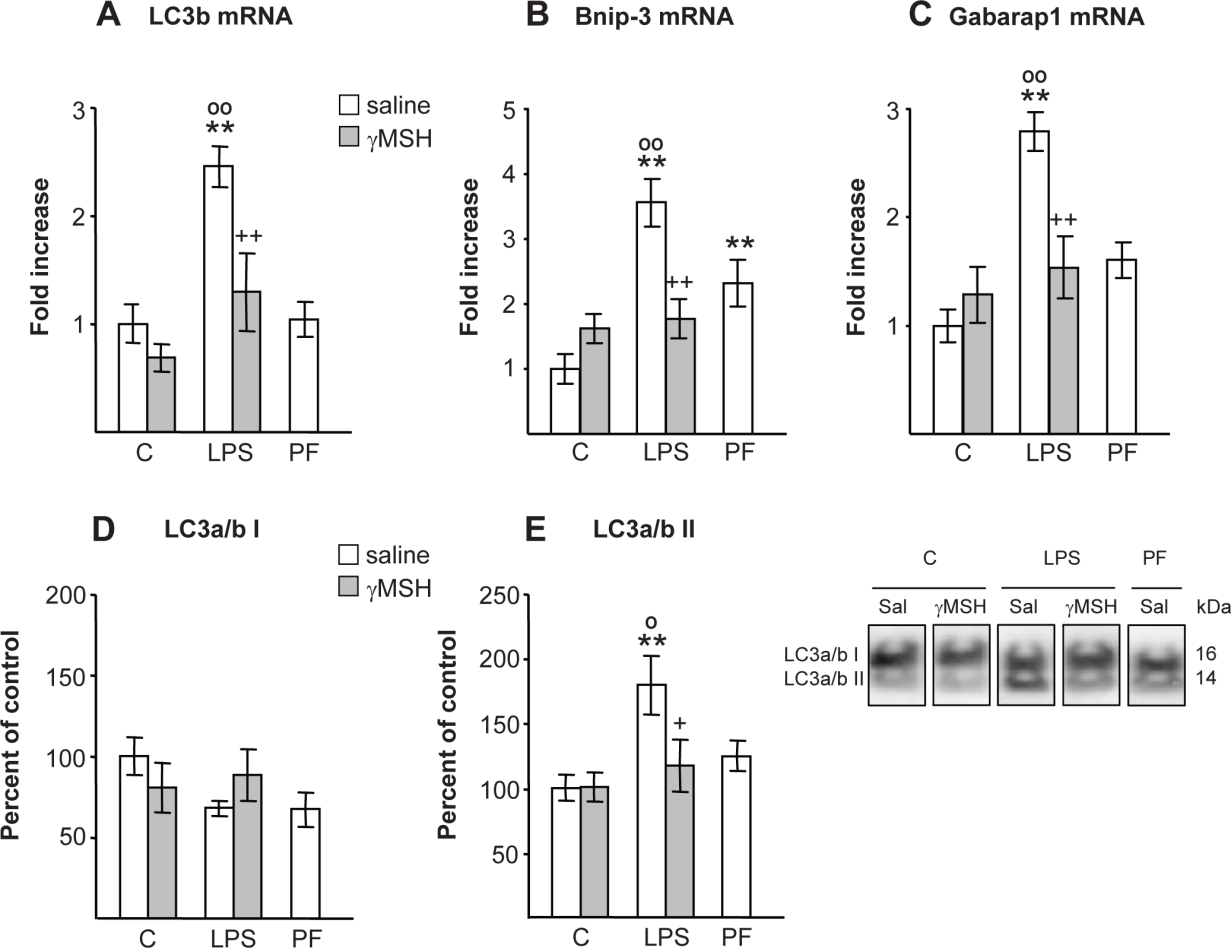
Effect of D-Trp(8)-γMSH (γMSH) treatment (500 μg/kg i.p.) on; autophagy-related marker LC3b mRNA (A), Bnip-3 (B) and Gabarap1 mRNA (C), on LC3a/b protein I and II (D and E), in *gastrocnemius* muscle of control rats and rats treated with LPS (250 μg/kg). PF = pair-fed rats. mRNA expression was quantified using real-time RT-PCR and is presented as increase of the mean value in control rats treated with saline. Proteins were measured by Western blotting and expressed as percentage of control rats treated with saline. Representative Western blots are shown at the bottom right. Boxes with immunoblots represent spliced images based on group and treatment order. LPS increased levels of LC3b, Bnip-3 and Gabarap1 mRNA and LC3a/b II protein (P<0.01) in rats treated with saline, but not in the group that received D-Trp(8)-γMSH. Results are expressed as means ± SE for 7–10 rats per group. **P< 0.01, vs. their respective control group. +P<0.05, ++P<0.01 vs. LPS-saline, °P<0.05, °°P<0.01 vs. PF. LSD multiple comparisons test, following one way ANOVA.

LPS injection induced *gastrocnemius* autophagic response, as indicated by the increase in expression of autophagy marker genes: LC3b, BCL2/adenovirus E1B 19 kDa protein-interacting protein 3 (Bnip-3) and gamma-aminobutyric acid receptor-associated protein (Gabarap1) (P<0.01, Fig 6A, 6B and 6C), as well as by the increased lipidation of LC3a/b protein. LPS did not significantly modify the protein LC3a/b I, but it increased the phospholipid-associated form LC3a/b II (P<0.01, Fig 6D and 6E). D-Trp(8)-γMSH administration prevented LPS-induced increase in LC3b, Bnip-3 and Gabarap1 mRNA (P<0.01) and in LC3a/b II protein (P<0.05). Pair feeding rats did not modify LC3b mRNA or phospholipid-associated form of the protein LC3a/b II, but increased Bnip-3 mRNA.

**Fig 7:**
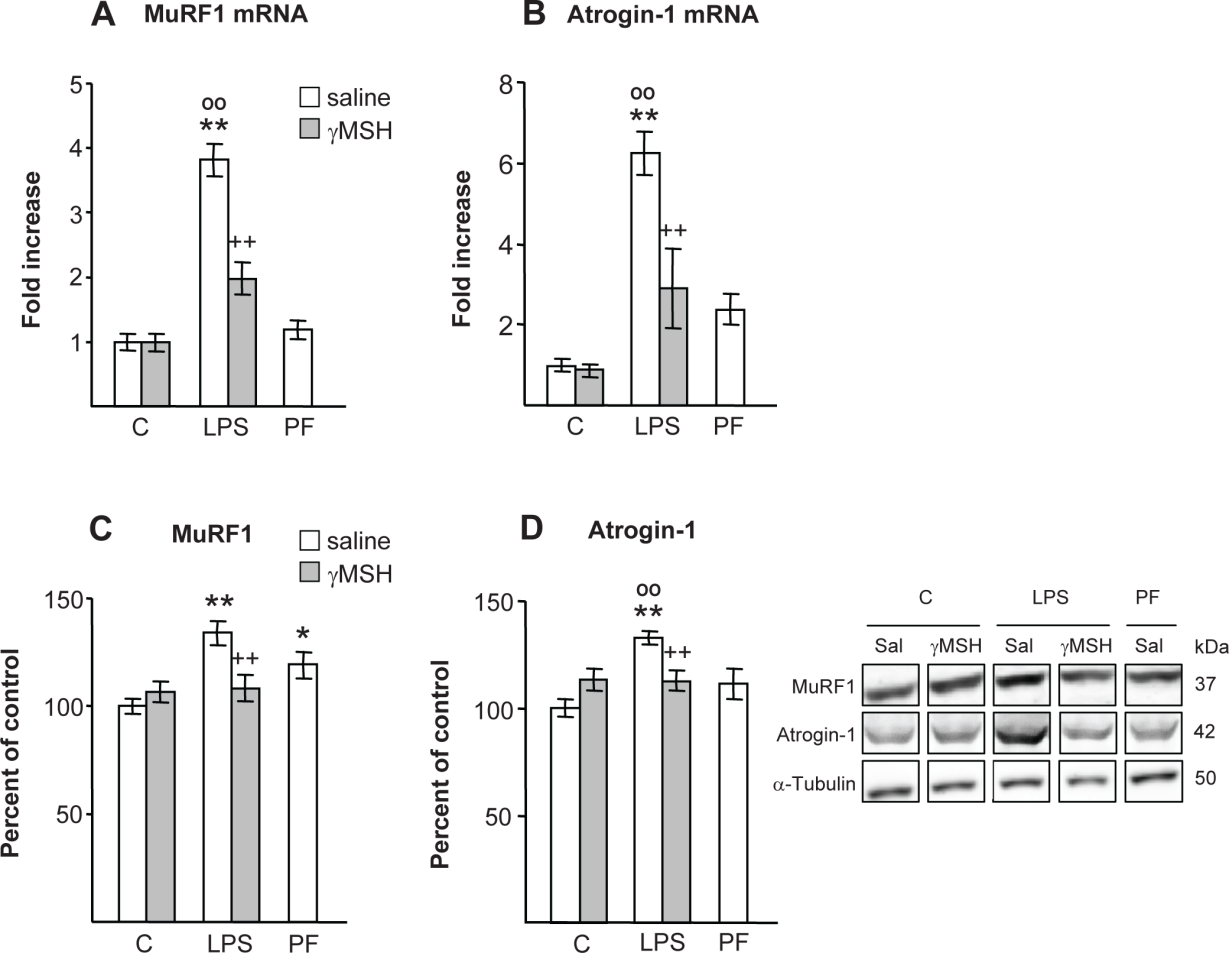
Effect of D-Trp(8)-γMSH (γMSH) treatment (500 μg/kg i.p.) on; MuRF1 mRNA and protein (A and C), and on atrogin-1 mRNA and protein (B and D), in *gastrocnemius* muscle of control rats and rats treated with LPS (250 μg/kg). PF = pair-fed rats. mRNA expression was quantified using real-time RT-PCR and is presented as increase of the mean value in control rats treated with saline. Proteins were measured by Western blotting and expressed as percentage of the control rats treated with saline. Representative Western blots are shown at the bottom right. Boxes with immunoblots represent spliced images based on group and treatment order. LPS increased levels MuRF1 and atrogin-1 proteins (P<0.01) in the rats treated with saline, but not in the group that received D-Trp(8)-γMSH. MuRF1 and atrogin-1 mRNAs were increased by LPS injection (P<0.01), and D-Trp(8)-γMSH attenuated these increases (P<0.01). Results are expressed as means ± SE for 7–10 rats per group. *P< 0.05 and **P< 0.01, vs. their respective control group. ++P<0.01 vs. LPS-saline, °°P<0.01 vs. PF. LSD multiple comparisons test, following one way ANOVA.

MuRF1 and atrogin-1 mRNA levels were significantly increased in response to LPS injection in saline treated rats (P<0.01, Fig 7A and 7B). D-Trp(8)-γMSH administration decreased LPS-induced increase in MuRF1 and atrogin-1 mRNA (P<0.01), where MuRF1 and atrogin-1 mRNA levels of these rats were similar to those of pair-fed rats, but higher than those of control rats treated with D-Trp(8)-γMSH (P<0.05). The protein expressions of MuRF1 and atrogin-1 were also higher after LPS injection (P<0.01, Fig 7C and 7D). Administration of D-Trp(8)-γMSH prevented the stimulatory effect of LPS on MuRF1 and atrogin-1 (P<0.01). Pair-fed rats had higher MuRF1 levels than control rats treated with saline (P<0.05).

### Myosin Heavy Chain expression (MHC)

**Fig 8:**
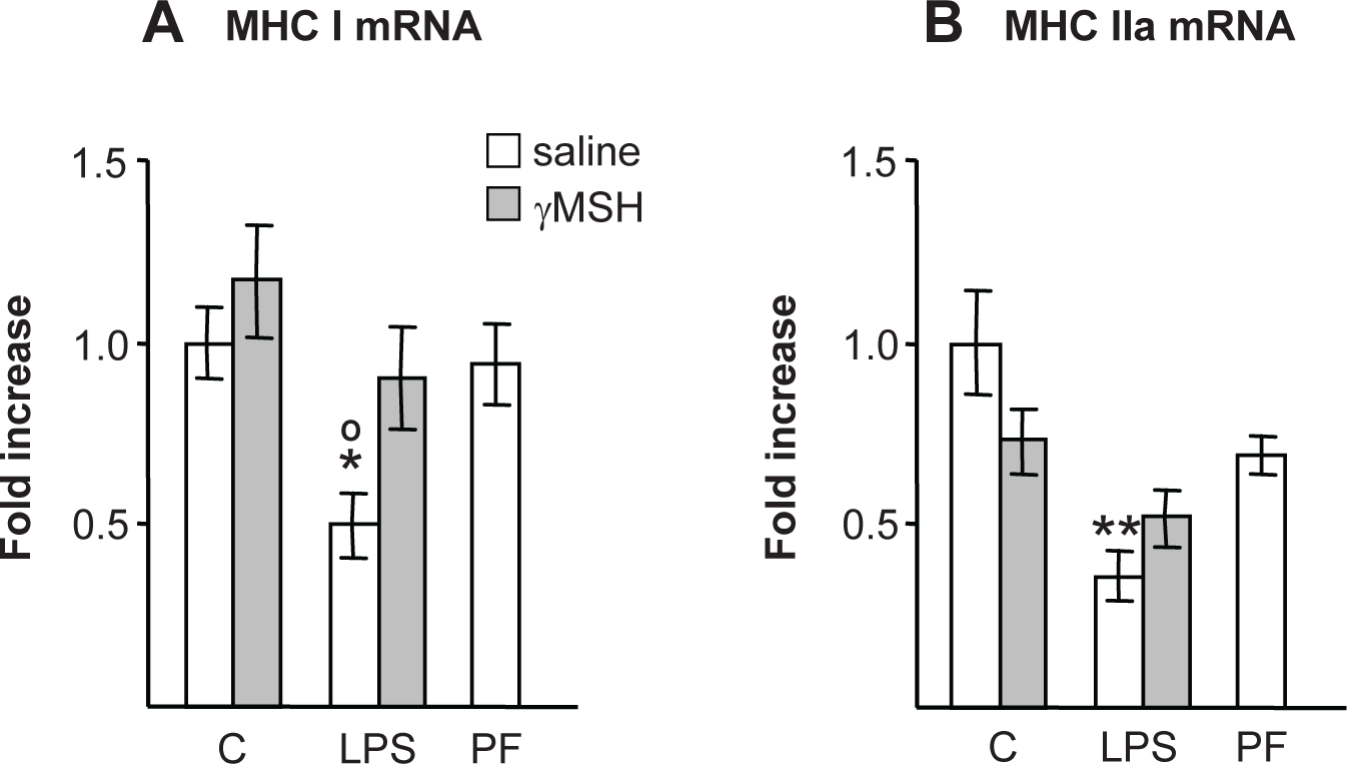
Effect of D-Trp(8)-γMSH (γMSH) treatment (500 μg/kg i.p.) on; MCH I (A) and MHC IIa mRNA (B), in *gastrocnemius* muscle of control rats and rats treated with LPS (250 μg/kg). PF = pair-fed rats. mRNA expression was quantified using real-time RT-PCR and is presented as the increase of the mean value in control rats treated with saline. LPS decreased *gastrocnemius* MCH I (P<0.05) and MCH II mRNA (P<0.01). The rats treated with D-Trp(8)-γMSH and LPS had MCH I and MCH II mRNA levels between those of control rats treated with D-Trp(8)-γMSH and rats treated with LPS alone. Data represent means ± SE (n = 7–9). *P< 0.05 and **P< 0.01, vs. their respective control group. °P<0.05 vs. PF. LSD multiple comparisons test, following one way ANOVA.

LPS injection decreased MHC types I (P<0.05) and IIa (P<0.01) in rats treated with saline (P<0.01, Fig 8A and 8B), but not in those treated with D-Trp(8)-γMSH. Control rats treated with D-Trp(8)-γMSH and pair-fed rats had lower MHC IIa values than those of control rats treated with saline, but differences were not significant.

### D-Trp(8)-γMSH was able to act directly on myotube cells

**Fig 9:**
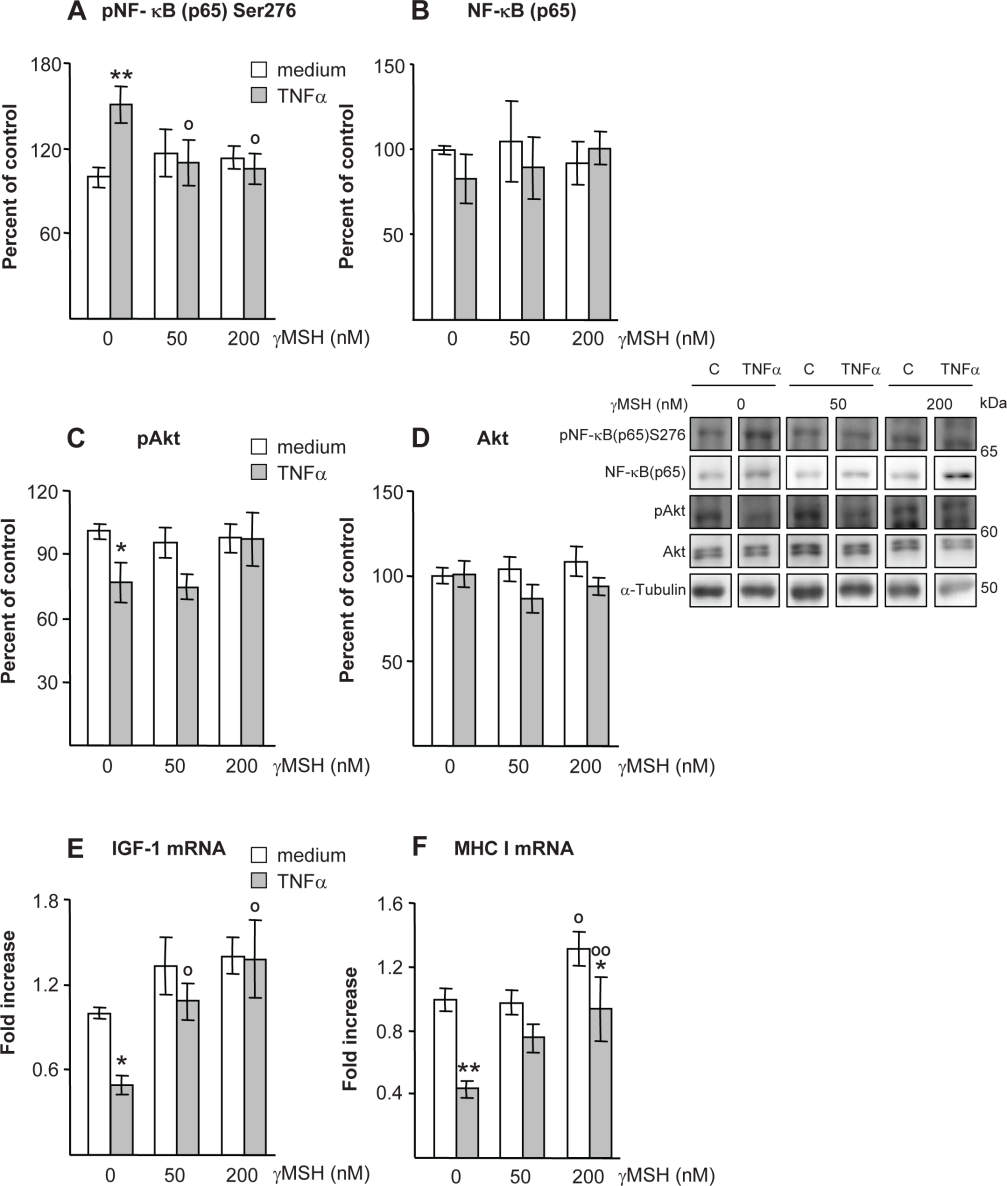
Effect of D-Trp(8)-γMSH (0, 50 or 200 nM) on; phospho-NF-κB(p65)Ser276 (A), NF-κB(p65) (B), phospho-Akt (C) Akt (D), IGF-I mRNA (E) and MHC I mRNA (F) in L6 myotubes cell cultures incubated with TNFα (10 μg/ml) or DMEM. Representative Western blots are shown at the middle right. Boxes with immunoblots represent spliced images based on group and treatment order. TNFα increased NF-κB(p65) phosphorylation (P<0.01) and decreased Akt phosphorylation (P<0.05), whereas D-Trp(8)-γMSH prevented those effects. IGF-I mRNA was decreased by TNFα (P<0.05), but not in the cells cultures with TNFα and D-Trp(8)-γMSH. TNFα also decreased MHC I mRNA (P<0.01) and D-Trp(8)-γMSH attenuated this effect. Data are expressed as mean ± SE for n = 6–8 wells per group, *P<0.05, **P<0.01 vs their respective myotube group incubated without TNFα, °P<0.05, °°P<0.01 vs their respective myotube group incubated without D-Trp(8)-γMSH. LSD multiple comparisons test, following one way ANOVA.

As shown in Fig 9, some of the effects of LPS on *gastrocnemius* could be observed in myotubes incubated with TNFα and D-Trp(8)-γMSH. Incubation with TNFα increased NF-κB(p65)Ser276 phosphorylation (P<0.01, Fig 9A), and decreased Akt phosphorylation (P<0.05, Fig 9C), whereas addition of D-Trp(8)-γMSH prevented those TNFα effects on myotubes.

IGF-I mRNA levels were decreased by TNFα (P<0.05, Fig 9E), whereas myotubes incubated with TNFα and 50 or 200 nM D-Trp(8)-γMSH had higher IGF-I expression than those incubated with TNFα alone. MHC I mRNA was also decreased by TNFα (P<0.01, Fig 9F). D-Trp(8)-γMSH, when added at the concentration of 200 nM, increased MHC I in both the myotubes incubated either with or without TNFα.

## Discussion

Administration of D-Trp(8)-γMSH was able to decrease inflammation and to attenuate the anorexigenic effect of endotoxin as well as the decrease in body weight. We have observed similar data after systemic administration of αMSH in rats injected with LPS [13]. These data suggest that those αMSH effects are mediated through MC3-R activation. In accordance with our data, it has been reported that peripheral D-Trp(8)-γMSH administration to normal mice acutely increases food intake [27], whereas MC3-RKO animals showed enhanced anorexia after LPS injection [20]. In addition, fasting-induced refeeding was blunted in the MC3-R-/- mouse [28]. In rats injected with LPS alone the systemic inflammatory response was also associated with increased IL-1β and COX-2 expression in the hypothalamus. Increased brain IL-1β levels have been reported as soon as 4 h after peripheral LPS challenge [29]. Systemic D-Trp(8)-γMSH administration was also able to decrease hypothalamic inflammation, since its administration prevented LPS-induced increase in hypothalamic IL-1β and COX-2 expression. The orexigenic action of D-Trp(8)-γMSH in rats injected with LPS might be related to its anti-inflammatory effect in the hypothalamus. In this sense, induction of COX-2 plays an important role in inflammatory anorexia [30, 31]. Therefore, it is possible that the inhibitory effect of D-Trp(8)-γMSH on LPS-induced anorexia is secondary to the decrease in hypothalamic COX-2 expression. In contrast to these data, chronic D-Trp(8)-γMSH treatment is unable to modify the anorexigenic effect of cancer [20]. Similarly, we have observed that chronic administration of D-Trp(8)-γMSH was unable to prevent both arthritis-induced anorexia and the increase in hypothalamic COX-2 expression, although D-Trp(8)-γMSH prevented arthritis-induced increase in hypothalamic IL-1β [21]. All these data suggest that acute MC3-R stimulation increases food intake by acting on hypothalamic COX-2, whereas this effect disappears with repeated daily systemic injections of the MC3-R agonist D-Trp(8)-γMSH, as it has previously been reported in normal mice [27].

In our data, D-Trp(8)-γMSH administration was able to decrease systemic inflammation, since it decreased LPS-induced increase in serum nitrites + nitrates as well as liver TNF and COX-2 expression. The elevated systemic inflammatory status after LPS injections was also reflected in *gastrocnemius* muscle, because NF-κB(p65) phosphorylation was increased. As we have reported in arthritic rats [21], D-Trp(8)-γMSH prevented NF-kB(p65) activation by LPS injection in the *gastrocnemius*. Furthermore, D-Trp(8)-γMSH is also able to prevent TNFα-induced NF-κB(p65) activation in myotubes. These data suggest that the anti-inflammatory effect of D-Trp(8)-γMSH on muscle cells can be exerted directly on skeletal muscle cells and it is not necessarily secondary to immune cell activation.

As it has previously been reported [6, 13, 32–34], LPS decreased Akt activation, increased FoxO1 and FoxO3 active protein, whereas the two complementary proteolytic pathways, ubiquitin-proteasome and autophagy, seem to be activated. D-Trp(8)-γMSH administration blocked LPS-induced alterations in Akt/FoxO signalling and downstream gene targets of FoxO1, FoxO3, atrogin-1, and MuRF1 in *gastrocnemius* muscle. LPS-induced increase in autophagic marker gene expression and in LC3a/b lipidation was also prevented by D-Trp(8)-γMSH. Taking into account that Akt/FoxO signalling represents a link between autophagy and the induction of MuRF1 and atrogin-1 [7], the effect of D-Trp(8)-γMSH on both proteolytic systems can be secondary to its action on NF-κB and Akt/FoxO signalling.

Administration of D-Trp(8)-γMSH prevented LPS-induced upregulation of the CRH-ACTH-corticosterone axis. Similarly, chronic D-Trp(8)-γMSH administration is also able to prevent arthritis-induced increase in ACTH and corticosterone [21]. Negative regulation of corticosterone release by MC3-R has previously been reported. MC3-R deficiency was found to produce mild hypercorticosteronemia [20, 28]. In addition, administration of γMSH prevents the stimulatory effect of IL-1β on corticosterone acting through central melanocortin receptors [35]. All these data suggest that activation of MC3-R prevents inflammation-induced glucocorticoid release. MC3-R and MC4-R are the only MC-R expressed highly in the brain [36], and both are activated by αMSH. However, αMSH treatment is unable to modify LPS-induced activation of the HPA axis [13]. Differences between αMSH and D-Trp(8)-γMSH effects could be explained by the fact that activation of brain MC4-R, contrary to MC3-R, has been shown to trigger activation of the hypothalamic-pituitary-adrenal (HPA) axis during stress [37]. In skeletal muscle glucocorticoids are potent inductors of proteolysis and in synergy with FoxO1, they directly transactivate MuRF1 and autophagy genes [8, 38, 39]. Furthermore, inhibition of glucocorticoid action by RU-486, an antagonist of the GC receptor, attenuates LPS-induced activation of autophagy and the ubiquitin-proteasome pathway and accelerated muscle proteolysis in sepsis [34]. Therefore, the protective effect of D-Trp(8)-γMSH treatment on *gastrocnemius* muscle proteolysis can be due, in part, to its effects on the hypothalamus-pituitary-adrenal axis.

It has been shown that LPS decreases circulating IGF-I and IGFBP-3 as well as their expression in the liver [23, 40, 41]. However, in the *gastrocnemius* IGF-I and IGFBP-3 expression are affected differently by LPS, where muscle IGF-I is decreased by endotoxin [4, 42], and IGFBP-3 is increased [13]. D-Trp(8)-γMSH treatment was able to prevent the effects of LPS on IGF-I levels, whereas it was unable to modify the IGFBP-3 response to LPS injections. The effect of LPS on IGF-I seems to be due, among other mechanisms, to a direct inhibitory action on liver cells [43], through the induction of COX-2 and iNOS [44, 45]. Taking into account that D-Trp(8)-γMSH had an anti-inflammatory effect in the liver, it is not surprising that it prevents the effects of LPS on serum and liver IGF-I levels. In addition to circulating IGF-I, muscle IGF-I also plays an important role in skeletal muscle physiology. It has been proposed that a deficit in muscle IGF-I is causally related to muscle wasting. In this sense, it has been reported that local IGF-I attenuates sepsis-induced *gastrocnemius* atrophy, by increasing muscle protein synthesis and potentially decreasing proteolysis [46]. Taking into account that LPS increases circulating TNFα and its expression in skeletal muscle [47], TNFα may contribute to the inhibitory effect of LPS on muscle IGF-I mRNA. In addition, an anti-TNFα antibody is able to prevent the LPS-induced reduction in IGF-I mRNA in rat skeletal muscle [48]. In our data, TNFα decreased MHC and IGF-I mRNA in L6 myotube cultures. These data are in accordance with those previously reported by Frost *et al*. [49]. As observed *in vivo*, D-Trp(8)-γMSH was able to prevent the inhibitory effect of TNFα on IGF-I mRNA in cultured myotubes. The effect of D-Trp(8)-γMSH in blocking the inhibitory of both LPS and TNFα-induced on IGF-I expression in muscle cells can be due to a direct effect on IGF-I gene. The blocking effect can also be mediated by its anti-inflammatory effect preventing NF-κB(p65) activation in myotubes or in the *gastrocnemius*.

In summary, in this article we report that D-Trp(8)-γMSH prevents LPS-induced anorexia, increased corticosterone levels and decreased IGF-I/Akt/mTOR signalling and muscle proteolysis. Our data also indicate that D-Trp(8)-γMSH exerts these anti-atrophic effects, at least in part, by inhibiting the LPS- or TNFα-dependent activation of NF-κB(p65) both *in vitro* and *in vivo*. The present study indicates that D-Trp(8)-γMSH is a molecule with potential therapeutic use for improving anorexia and muscle wasting during sepsis.
